# Resilient Multi-Robot Coverage Path Redistribution Using Boustrophedon Decomposition for Environmental Monitoring

**DOI:** 10.3390/s24237482

**Published:** 2024-11-23

**Authors:** Junghwan Gong, Hyunbin Kim, Seunghwan Lee

**Affiliations:** School of Electronic Engineering, Kumoh National Institute of Technology, Gumi 39177, Republic of Korea; 20236005@kumoh.ac.kr (J.G.); guswkd159@kumoh.ac.kr (H.K.)

**Keywords:** boustrophedon decomposition, multi-robot coverage path planning, propagation, multi-robot systems

## Abstract

This study introduces a resilient and adaptive multi-robot coverage path planning approach based on the Boustrophedon Cell Decomposition algorithm, designed to dynamically redistribute coverage tasks in the event of robot failures. The proposed method ensures minimal disruption and maintains a balanced workload across operational robots through a propagation-based redistribution strategy. By iteratively reallocating the failed robot’s coverage path to neighboring robots, the method prevents any single robot from becoming overburdened, ensuring efficient task distribution and continuous environmental monitoring. Simulations conducted in five distinct environments, ranging from simple open areas to complex, obstacle-rich terrains, demonstrate the method’s robustness and adaptability. A key strength of the proposed approach is its fast and efficient task reallocation process, achieved with minimal propagation cycles, making it suitable for real-time applications even in complex scenarios. The approach reduces task variance and maintains balanced coverage throughout the mission.

## 1. Introduction

The deployment of multi-robot systems has experienced significant growth across a wide range of applications, including large-scale surveillance [1], agricultural automation [2], search and rescue operations [3], geophysical surveys, and environmental monitoring [4,5,6,7]. This growing trend is largely due to the inherent scalability, efficiency, and ability of multi-robot systems to cover extensive and complex environments more effectively than single-robot systems. As these systems advance in sophistication, the need for more refined and adaptive coverage path planning (CPP) methods becomes increasingly critical, particularly for missions requiring extended, uninterrupted monitoring, such as environmental conservation and infrastructure inspection [5,8]. The primary challenge faced by multi-robot systems is maintaining mission continuity in the face of unforeseen circumstances such as hardware malfunctions, robot failures, or power depletion [1,9]. The incapacitation of a robot can create significant gaps in coverage, which may jeopardize mission success. Recent studies have emphasized the importance of addressing these issues through dynamic path replanning and task redistribution among the remaining functional robots.

In this paper, we propose a novel approach to multi-robot coverage path planning designed to address these challenges. Our method, based on the Boustrophedon Cell Decomposition (BCD) algorithm [10], introduces dynamic task reassignment and energy-aware path adjustments. These enhancements allow the robots to autonomously redistribute their responsibilities in the event of a failure or low battery, minimizing the impact on overall mission performance. The key contributions of this paper are twofold:

- We introduce a task redistribution framework, applying the concept of propagation to BCD-based multi-robot coverage technology, ensuring balanced coverage even in the event of robot failure, and propose a new algorithm.
- We validate the performance of our approach through simulations in various environments, ranging from obstacle-rich to open spaces, and demonstrate that the propagation process leads to equalized task distribution over time.

## 2. Related Work

CPP plays a critical role in multi-robot systems, particularly in domains such as environmental monitoring, search and rescue, and agricultural automation, where complete area coverage is essential. One of the seminal methods in CPP is the BCD algorithm, introduced by Choset et al. [10]. The BCD algorithm is a grid-based path planning method that divides the workspace into discrete cells, allowing robots to cover each cell in a systematic, back-and-forth motion. This boustrophedon motion effectively manages complex environments by minimizing unnecessary turns, making it particularly advantageous for structured coverage tasks. In addition, this method guarantees full coverage in structured environments and has been widely adopted due to its computational efficiency. However, classical CPP methods [11,12,13], including BCD [14], often struggle in dynamic environments, where unexpected robot failures or environmental changes may occur.

Gabriely et al. [15] utilized a spanning tree to systematically guide a single robot’s movements, ensuring full coverage of the target area. However, the method is primarily designed for a single robot, which limits its scalability and adaptability in dynamic multi-robot systems. Tang et al. [16] designed the MSTC* algorithm for multi-robot coverage path planning to operate under physical constraints such as limited robot maneuverability and environmental obstacles. Additionally, Tang et al. [17] introduced a large-scale multi-robot coverage path planning method using a local search algorithm to optimize task allocation and path efficiency. Their approach addresses scalability by focusing on localized improvements in robot paths, enhancing overall coverage performance. In structured environments, Lu et al. [18] proposed the TMSTC* algorithm, which minimizes unnecessary turns, reduces energy consumption, and increases efficiency. While these methods perform well under certain conditions, in scenarios where all points within an area must be sufficiently visited, such as in cleaning tasks, BCD-based methods remain widely used due to their efficiency. Kapoutsis et al. [19] introduced the DARP algorithm, which optimally divides the target environment into regions to ensure balanced and efficient coverage. Similarly, Karapetyan et al. [20] developed an efficient multi-robot coverage strategy for known environments, focusing on minimizing redundant coverage and improving coordination. Sun et al. [11] applied genetic algorithms for multi-robot path planning to ensure complete coverage and optimal task distribution through iterative refinement. For specific applications like geophysical surveys, Azpúrua et al. [21] proposed a method based on hexagonal segmentation, which ensures uniform coverage and minimizes overlap. Huang et al. [22] addressed the unique challenges of environments with multiple land cover types by designing a CPP algorithm that adapts to terrain characteristics. The comprehensive survey in [4] categorized CPP methods and highlighted the growing need for robustness in multi-robot systems, particularly regarding task allocation and energy efficiency, which are critical for large-scale missions. In surveillance systems, Gong et al. [23] proposed hierarchical area-based and path-based heuristic approaches that enhance scalability and adaptability, optimizing coverage in both simple and complex environments. While these studies focus on optimizing coverage efficiency, for long-term missions, it is also essential to incorporate fault-tolerant mechanisms, as in our study, ensuring balanced task assignment and resilience to robot failures.

As multi-robot systems became more sophisticated, researchers began focusing on dynamic and fault-tolerant solutions. Zhou et al. [24] proposed a reactive task allocation and path planning framework for heterogeneous multi-robot teams, including quadrupedal and wheeled robots. When robots encounter disturbances, the framework employs local and global reallocation strategies, allowing tasks to be completed without entirely replanning. Fazli et al. [25] introduced a method for multi-robot repeated area coverage, emphasizing task partitioning and workload balance in dynamic environments. Their method ensures that coverage tasks are consistently distributed among robots, even in the event of a robot failure. Building upon this, our work introduces a fault-tolerant mechanism that redistributes tasks dynamically, maintaining balanced coverage even as robots experience failures. Song et al. [9] presented the CARE framework, enhancing resilience in multi-robot systems by redistributing tasks adaptively during failures in unknown environments. While CARE focuses on unknown environments, our approach employs BCD for structured path planning and dynamic task redistribution, ensuring adaptability and balanced coverage across diverse terrains. Although CARE’s approach differs slightly from ours, its redistribution methodology represents a recent and relevant study, making it a suitable benchmark for comparison [1]. CARE is a representative CPP study that responds when a robot problem occurs and was chosen as a comparison group to validate the efficient and balanced coverage performance of the proposed study. Lee [1] made a significant contribution by developing a real-time coverage area reassignment strategy for multi-robot surveillance systems, dynamically redistributing the failed robot’s coverage area to minimize mission disruption. Our research extends the real-time reassignment strategy [1] by integrating it into a BCD-based framework, enabling both structured coverage and real-time failure handling. Rekleitis et al. [14] also focused on BCD for multi-robot coverage, primarily addressing coordination to avoid redundant coverage. However, their approach did not account for dynamic failures, which our method addresses by incorporating task redistribution to ensure no coverage gaps occur due to robot failures. Scalability remains a challenge in large-scale missions, and Collins et al. [26] tackled this by dividing complex environments into smaller regions for each robot, enabling efficient coverage in non-convex areas. Our approach builds on this by adding real-time fault tolerance, dynamically reassigning tasks when robots fail. Other specialized CPP methods such as the method described in [2] for agricultural automation and Cai et al.’s [8] method for maritime search and rescue, further highlight the need for adaptability in specific domains. We build on these methods by integrating a fault-tolerant mechanism to ensure robust and efficient coverage across various environmental conditions. In terms of 3D environments, Almadhoun et al. [27] introduced a hybrid CPP approach for volumetric coverage, particularly useful in geophysical surveys or 3D mapping. The integration of fault-tolerant features in multi-robot systems continues to be a major research focus. For example, Bähnemann [28] revisited BCD, incorporating the Generalized Traveling Salesman Problem (GTSP) to optimize path length and coverage efficiency. However, their method did not address robot failures directly, which is a critical focus of our work.

## 3. Problem Description

In multi-robot coverage tasks, ensuring efficient and balanced coverage of large-scale environments becomes a critical challenge, particularly when unexpected robot failures occur during mission execution. This study addresses the problem of redistributing the coverage responsibility of a failed robot among the remaining operational robots, ensuring that the mission objectives are maintained without significant disruptions or inefficiencies.

Consider a multi-robot system consisting of *N* autonomous robots $R=\{R_{1},R_{2},...,R_{N}\}$ operating in a given environment *M*. Each robot is initially assigned a coverage path generated using a decomposition-based algorithm, such as the BCD method, ensuring efficient and complete coverage of the environment. Each robot’s path consists of multiple feature nodes that the robot must visit to cover its assigned area. When a robot $R_{k}$ becomes non-operational due to factors such as hardware malfunctions or battery depletion, a coverage gap arises in its previously assigned path. The primary problem then is to redistribute the coverage tasks of the failed robot among the remaining N−1 robots in a way that minimizes imbalance and ensures efficient completion of the mission. This redistribution must be handled in a manner that avoids overburdening any single robot and maintains overall system efficiency.

### 3.1. Key Assumptions and Requirements

In addressing the problem of balanced MCPP, several key assumptions and requirements underpin the proposed method, ensuring its applicability and effectiveness in real-world scenarios. These assumptions and requirements are summarized as follows:

- It is assumed that the BCD algorithm generates collision-free and non-overlapping paths for each robot. The paths are divided based on the sensing range $R_{S}$ of the robots, ensuring that each robot can efficiently cover its assigned area.
- Robots may experience unexpected failures during the mission, and these failures result in unmonitored areas. The failed robot $R_{k}$ has neighboring robots $R_{N}^{L_{1}}$ capable of absorbing the additional coverage tasks.
- These neighboring robots are determined based on their spatial proximity to $R_{k}$, and they form an adjacency structure that facilitates the redistribution process.
- Redistribution aims to minimize the variance in the workload across the remaining robots. This involves ensuring that the additional coverage responsibilities are allocated in proportion to each robot’s current task load and their proximity to the failed robot’s path.
- It is assumed that each robot can dynamically adjust its path to absorb new coverage tasks without significantly deviating from its original path plan.
- The proposed solution leverages a propagation-based approach [1], where redistribution starts with the nearest neighboring robots and progressively extends to more distant robots if necessary. This ensures that the additional workload is not concentrated on a single robot, maintaining a balanced task distribution.
- It is assumed that the robots have a reliable communication mechanism to share coverage information, allowing them to coordinate effectively during the redistribution process. This communication is crucial for ensuring that the coverage paths are adjusted in a synchronized manner across the entire team.

### 3.2. Objective

The primary objective of this study is to develop a robust and adaptive method for redistributing coverage tasks when a robot fails, ensuring that the remaining robots can continue the mission without significant interruptions. The proposed method must maintain a balanced task distribution, minimize the overall coverage time, and adapt to dynamic changes in robot availability. By considering these assumptions and focusing on an iterative, propagation-based redistribution strategy, this study aims to develop a comprehensive solution that allows multi-robot systems to handle failures efficiently, ensuring that mission objectives are achieved even in dynamic and uncertain operational environments. This sets the foundation for the detailed methodology discussed in the following section, where the proposed propagation-based region allocation and path replanning strategy are implemented to address the problem effectively.

## 4. Proposed Method

**Figure 1:**
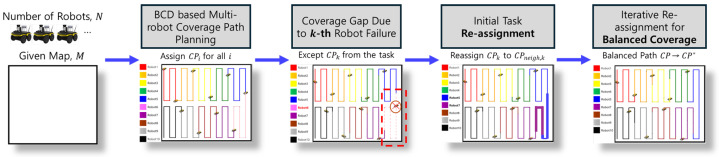
Flowchart of the proposed method.

The proposed multi-robot path redistribution method is grounded in the BCD algorithm, where the coverage path of a failed robot is adaptively redistributed to the remaining robots using an iterative reassignment strategy. Figure 1 illustrates the flowchart of the proposed method, which comprises five main stages. Initially, the environment map and the number of robots are provided, with the map being either derived from an SLAM (simultaneous localization and mapping) process or simplified and approximated as a polygonal representation. The BCD algorithm is then applied to generate multi-robot coverage paths that effectively divide the overall environment among all robots. In the event of a robot failure, a coverage gap emerges, as shown in the second stage of the flowchart. The proposed method then enters the third stage, where an initial task reassignment takes place to redistribute the failed robot’s coverage path segments to the nearest robots. The fourth stage involves iterative reassignment, where the remaining robots adjust their paths through propagation to ensure a balanced coverage of the environment. This process continues until the propagation is complete. As a result, the proposed method ensures that the overall mission objectives are maintained efficiently, despite the dynamic changes in robot availability.

### 4.1. MCPP Based on BCD

The first step of the proposed method involves conducting MCPP using the BCD algorithm. This approach enables efficient coverage by dividing the given environment into multiple polygonal cells, represented by the set $P=\{P_{1},P_{2},\ldots ,P_{k}\}$, where *k* denotes the total number of cells generated by the BCD algorithm. Within each of these cells, boustrophedon paths are established while ensuring that each path remains connected and collision-free, even in the presence of multiple obstacles. By examining potential collision points, the spacing between paths within each cell is adjusted according to the robot’s sensing range $R_{S}$, ensuring efficient coverage without redundant overlaps.

For MCPP involving *N* robots, the entire path set *P* is evenly divided among the robots to form *N* distinct coverage paths. The resulting paths allow each robot to effectively cover a specific portion of the environment. The overall coverage path $CP_{i}$ for the *i*-th robot is derived using the following equation:

(1) $$CP_{1:N}=\mathrm{BCD}\left(M,N,R_{S}\right),$$

where *M* denotes the map representing the environment. *N* is the total number of robots and $R_{S}$ is the sensing distance used to adjust the path spacing within each cell. The coverage path $CP_{i}$ allocated to the *i*-th robot consists of an ordered set of feature nodes that the robot must visit during the coverage task:

(2) $$CP_{i}=\left\{cp_{i,1},cp_{i,2},\ldots ,cp_{i,m_{i}}\right\},$$

where $m_{i}$ represents the total number of feature nodes assigned to the *i*-th robot. These feature nodes vary for each robot, depending on the complexity of the cell structure and the number of turning points along their respective paths. The length of each robot’s coverage path $LP_{i}$ is a critical parameter, computed as the sum of Euclidean distances between consecutive feature nodes along the path:

(3) $$LP_{i}=\sum_{j=1}^{m_{i}-1}{∥cp_{i,j}-cp_{i,j+1}∥}_{2},$$

where ${∥\cdot ∥}_{2}$ is the Euclidean norm between two consecutive nodes $cp_{i,j}$ and $cp_{i,j+1}$. This ensures that the length of the coverage path accurately reflects the distance traveled by the robot. To further enhance coordination among robots, it is essential to consider the inter-robot distances. The distance between two robots $R_{i}$ and $R_{j}$, denoted as $\mathrm{dist}(R_{i},R_{j})$, is defined as the minimum Euclidean distance between all pairs of nodes from their respective coverage paths $CP_{i}$ and $CP_{j}$. This inter-robot distance is calculated as follows:

(4) $$\mathrm{dist}\left(R_{i},R_{j}\right)=\min_{\begin{array}{c} cp_{i,a}\in CP_{i}\\ cp_{j,b}\in CP_{j} \end{array}}{∥cp_{i,a}-cp_{j,b}∥}_{2},$$

where $cp_{i,a}$ and $cp_{j,b}$ represent individual nodes from the coverage paths $CP_{i}$ and $CP_{j}$, respectively. The distance function $\mathrm{dist}(R_{i},R_{j})$ is vital for determining the proximity between robots, which plays a crucial role when dynamically redistributing the coverage path of a failed robot to its neighboring robots. This ensures efficient path reallocation while maintaining balanced coverage throughout the multi-robot system. By integrating the BCD algorithm’s coverage path planning with these distance calculations, the proposed method ensures that each robot covers its designated area optimally while being capable of adapting to dynamic changes, such as robot failures, ensuring a comprehensive and balanced multi-robot coverage strategy.

### 4.2. Tree Construction for the Excluded Robot

**Figure 2:**
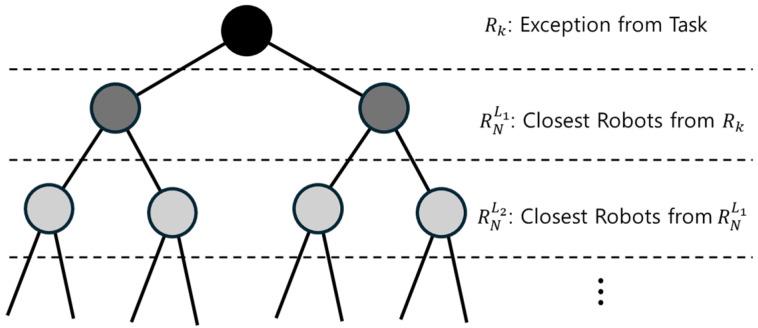
Illustration of the adjacency tree used for path redistribution among neighboring robots when a robot, $R_{k}$, failure occurs.

When a robot $R_{k}$ becomes incapacitated due to factors such as battery depletion or hardware malfunction, a coverage gap appears in its previously assigned path $CP_{k}$. To effectively resolve this and redistribute the uncovered area to other operational robots, an adjacency tree, *T*, is constructed with $R_{k}$ as the root node. As shown in Figure 2, the robots nearest to the excluded robot $R_{k}$, denoted as $R_{N}^{L_{1}}$, form the first level $L_{2}$ of the adjacency tree. Subsequently, robots closest to those in level $L_{2}$ that have not yet been incorporated into the tree structure form the next level, $L_{3}$. This hierarchical process continues, with each successive level consisting of the nearest robots not already included, until all relevant neighboring robots are accounted for. The total number of levels in the tree depends on the configuration and distribution of the coverage paths within the given environment.

### 4.3. Propagation-Based Coverage Redistribution and Path Replanning

The core idea of the proposed method is to redistribute the coverage path $CP_{k}$ of the excluded robot $R_{k}$ to its neighboring robots $R_{N}^{L_{1}}$ using a propagation-based approach, particularly in BCD-based CPP. A straightforward method might involve dividing $CP_{k}$ equally among the robots in $R_{N}^{L_{1}}$ based on their number, $|R_{N}^{L_{1}}|$. However, this simple division often results in an imbalanced task distribution, as certain robots, especially those closest to the excluded robot, may inherit a disproportionate workload. In this study, task allocation is based on the length of the coverage path, and the balance of the workload is measured using the variance $\sigma_{\mathrm{balancing}}$, which is computed as follows:

(5) $$\sigma_{\mathrm{balancing}}=\frac{\sum_{j=1,j\neq k}^{N}{\left(LP_{j}-\bar{LP}\right)}^{2}}{N-1},$$

(6) $$\bar{LP}=\frac{\sum_{j=1,j\neq k}^{N}LP_{j}}{N-1},$$

where $\bar{LP}$ represents the mean coverage path length across all operational robots, excluding $R_{k}$.

To minimize $\sigma_{\mathrm{balancing}}$, a propagation-based strategy is required, which is particularly well suited for handling boustrophedon-like coverage paths. Consider the *i*-th robot among the set $R_{N}^{L_{1}}$. Suppose this robot has a subtree size $N_{i\in L_{1}}$ relative to the total N−1 operational robots. The allocation ratio $\alpha_{i,\mathrm{assign}}$ for this robot is then determined by

(7) $$\alpha_{i,\mathrm{assign}}=\frac{N_{i\in L_{1}}+1}{N-1},$$

where the numerator $N_{i\in R_{N}^{L_{1}}}+1$ represents the number of child robots included in the subtree of the robot, along with the robot itself, compared to the total number N−1.

Using this allocation ratio $\alpha_{i,\mathrm{assign}}$, each robot in $R_{N}^{L_{1}}$ inherits a portion of the coverage path from $CP_{k}$ proportional to $\alpha_{i,\mathrm{assign}}$. For example, if N=11 and the *i*-th robot has a subtree size of 5, then $\alpha_{i,\mathrm{assign}}=\frac{6}{10}=0.6$. Applying this ratio, the total coverage path length $L_{i}$ of the *i*-th robot is updated as follows:

(8) $$L_{i}=L_{i}+\alpha_{i,\mathrm{assign}}\cdot L_{k},$$

where $L_{k}$ represents the length of the excluded robot’s coverage path. Due to the characteristics of the BCD method, the size of $R_{N}^{L_{1}}$, denoted as $|R_{N}^{L_{1}}|$, is typically less than 2. Additionally, the extra path segments can be conveniently assigned to the nearest robots by extending the endpoints of their existing coverage paths. This adjustment primarily involves modifying the endpoints $cp_{i,1}$ or $cp_{i,m_{i}}$, which are closest to $CP_{k}$, to accommodate the reassigned coverage.

After this initial reassignment, the robots within $R_{N}^{L_{1}}$ experience an unbalanced increase in their coverage paths. To address this imbalance, a propagation strategy is employed iteratively, wherein child robots of those in $R_{N}^{L_{1}}$ adjust their coverage path lengths according to the following equation:

(9) $$L_{\mathrm{child}}=L_{\mathrm{child}}+\alpha_{\mathrm{child},\mathrm{assign}}\cdot L_{k},$$

(10) $$\alpha_{\mathrm{child},\mathrm{assign}}=\frac{N_{\mathrm{child}}+1}{N-1},$$

where $L_{\mathrm{child}}$ represents the coverage path lengths of the child in the adjacency tree. The term $\alpha_{\mathrm{child},\mathrm{assign}}$ represents the allocation ratio with $N_{\mathrm{child}}$ being the cardinality (size) of the subtree rooted at the child node. The product $\alpha_{\mathrm{child},\mathrm{assign}}\cdot L_{k}$ determines how much of the parent’s coverage path the child robot should inherit. Consequently, the parent’s coverage path length should be reduced as follows:

(11) $$L_{\mathrm{parent}}=L_{\mathrm{parent}}-\alpha_{\mathrm{child},\mathrm{assign}}\cdot L_{k}.$$

As the propagation progresses through the levels of the adjacency tree, $L_{\mathrm{child}}$ and $L_{\mathrm{parent}}$ are iteratively updated until all robots corresponding to the leaf nodes have received their adjusted coverage paths. For example, the *j*-th node, which is a child node, has its path $CP_{j}$ extended towards its parent’s coverage path according to $\alpha_{j,\mathrm{assign}}$. However, in subsequent iterations, this node becomes a parent node, and its path will be reduced as its own child node’s coverage path increases. The final updated coverage path for the *j*-th node is denoted as $CP_{j}^{\prime }$. Algorithm 1 outlines the process of redistributing the coverage path of a failed robot $R_{k}$ to its neighboring robots using a propagation-based approach within the context of BCD-based CPP. The algorithm begins by taking as input the set of coverage paths CP, the total number of operational robots *N*, and the failed robot $R_{k}$. It constructs an adjacency tree *T*, where $R_{k}$ serves as the root node, and the neighboring robots $R_{N}^{L_{1}}$ form the first level of the tree. Next, for each robot $i\in R_{N}^{L_{1}}$, the algorithm calculates an allocation ratio $\alpha_{i,\mathrm{assign}}$ based on the size of the robot’s subtree. This ratio determines the portion of the failed robot’s coverage path that will be inherited by robot *i*. The robot’s coverage path is then updated accordingly. Following the initial redistribution to the robots in $R_{N}^{L_{1}}$, the algorithm iteratively propagates the remaining coverage path further down the hierarchy. For each robot in subsequent levels of the adjacency tree, similar calculations are performed to allocate and update the coverage paths of child robots and their corresponding parent robots. The propagation process continues until all robots in the tree have received their adjusted coverage paths, ensuring a balanced distribution of the failed robot’s workload. The final result is the updated set of coverage paths $CP^{\prime }$, which the Algorithm 1 returns.
**Algorithm 1** Propagation-Based Coverage Redistribution1:**Input:** Coverage paths CP, *N*, Failed robot $R_{k}$2:**Output:** Updated coverage paths $CP^{\prime }$3:Parent ←∅4:Construct the adjacency tree *T* based on $R_{k}$5:**for** each child robot $i\in R_{N}^{L_{1}}$ **do**6:    Calculate $\alpha_{i,\mathrm{assign}}$ from (7)7:    Calculate $L_{i}$ from (8)8:    $CP_{i}^{\prime }\leftarrow \mathrm{UpdatePaths}\left(CP_{i},L_{i},\alpha_{i,\mathrm{assign}}\right)$9:    Parent ←Parent∪{i}10:**end for**11:**while** Parent ≠∅ **do**12:    j←Extract(Parent)13:    Child ←ExtractChild(T,j)14:    Calculate $\alpha_{\mathrm{Child},\mathrm{assign}}$ from (10)15:    Calculate $L_{\mathrm{Child}}$ from (9)16:    Calculate $L_{j}$ from (11)17:    $CP_{\mathrm{Child}}^{\prime }\leftarrow \mathrm{UpdatePaths}\left(CP_{\mathrm{Child}},L_{\mathrm{Child}},\alpha_{\mathrm{Child},\mathrm{assign}}\right)$18:    $CP_{j}^{\prime }\leftarrow \mathrm{UpdatePaths}\left(CP_{j}^{\prime },L_{j},\alpha_{\mathrm{Child},\mathrm{assign}}\right)$19:    Parent ←Parent∪{Child}20:    Parent ←Parent∖{j}21:**end while**22:**return** Updated paths $CP^{\prime }$

## 5. Simulation

### 5.1. Simulation Environments

**Figure 3:**
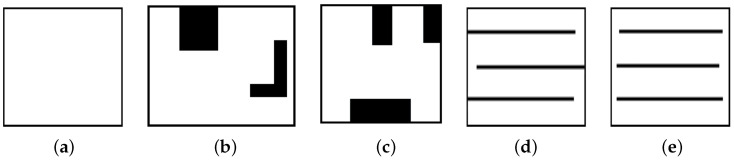
Simulation environments. (**a**) Map 1 represents a simple open area. (**b**) Map 2 and (**c**) Map 3 show areas with multiple obstacles. In addition, (**d**) Map 4 and (**e**) Map 5 represent areas with multiple lanes.

In this study, five different environments were used to evaluate the proposed MCPP and redistribution method, as shown in Figure 3. These environments vary in complexity and coverage challenges, providing a comprehensive test of the algorithm’s performance. Figure 3a represents a simple environment without obstacles, assessing basic coverage efficiency. Figure 3b,c introduce two and three obstacles, respectively, allowing us to evaluate how the algorithm handles obstacle avoidance and path redistribution. Figure 3d,e contain multiple lanes, testing the algorithm’s ability to balance coverage across segmented regions, relevant in scenarios like agriculture or warehouse management. These five simulation environments provide a diverse set of challenges to thoroughly assess the adaptability, efficiency, and robustness of the multi-robot coverage path replanning methods. All robots in the simulation operate at the same travel speed and feature 360-degree omnidirectional navigation. Additionally, with holonomic motion capabilities, the robots are not limited by a turning radius, allowing for greater maneuverability. By evaluating the algorithm across these varied conditions, we demonstrate its capability to handle real-world complexities and ensure reliable performance in different types of environments.

### 5.2. Simulation Results: Multi-Robot Coverage Path Planning

**Figure 4:**
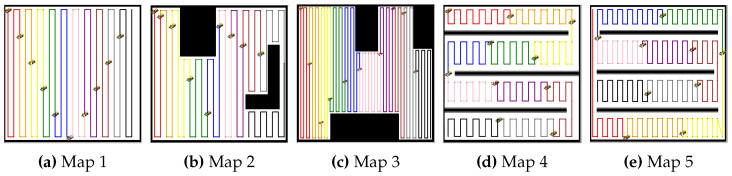
BCD-based MCPP results for 10 robots. (**a**–**e**) represent the results of the MCPP for each map.

**Figure 5:**
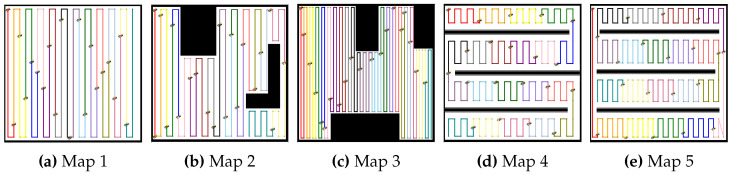
BCD-based MCPP results for 20 robots. MCPP results for 10 robots. (**a**–**e**) represent the results of the MCPP for each map.

The simulation results provide a comprehensive evaluation of the proposed MCPP and redistribution method across five distinct environments. First, the BCD-based MCPP was performed to assign coverage tasks to all robots. Figure 4 and Figure 5 show the coverage path planning results for 10 robots and 20 robots, respectively. Figure 4a shows the coverage paths for 10 robots in an open environment with no obstacles. The BCD algorithm efficiently divides the area among the robots, ensuring minimal overlap. In Figure 4b, two obstacles are introduced, and the algorithm maintains balanced coverage across the area, demonstrating its ability to allocate tasks even in more complex environments. Figure 4c further adds multiple obstacles, highlighting the algorithm’s ability to adjust coverage distribution in increasingly complex settings. Figure 4d,e depict lane-based environments, where the BCD algorithm ensures balanced and efficient coverage across the segmented lanes, applicable in structured environments such as warehouses or agricultural fields. Similarly, Figure 5 presents the results for 20 robots. In Figure 5a, the open environment is covered efficiently, even with the increased number of robots. Figure 5b,c introduce obstacles, and the algorithm maintains efficient and balanced coverage. Figure 5d,e show the lane-based environments, where the algorithm continues to manage segmented areas effectively, ensuring minimal overlap and maintaining balanced coverage tasks across the lanes.

### 5.3. Simulation Results: Balanced Path Redistribution

The simulation results offer a comprehensive evaluation of the proposed MCPP and redistribution method across five distinct environments. These results highlight how the proposed method effectively adapts to various challenges, maintaining efficient and balanced coverage even in the event of robot failures. The performance of the proposed method is also compared to a method that applies only initial propagation, as well as a similar approach to [9], which considers only adjacent neighbors.

#### 5.3.1. Simple Open Environment (Map 1)

**Figure 6:**
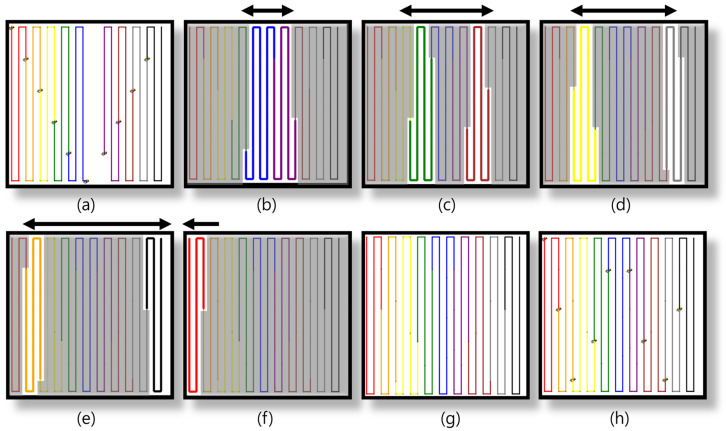
Task exclusion and propagation progress. In (**a**), the failure of a robot is shown. (**b**–**h**) depict the sequence of the propagation process.

Figure 6 illustrates the sequence of propagation stages following the failure of a robot in a simple open environment. In Figure 6a, the failed robot is excluded (represented as a blank space), resulting in a coverage gap. Figure 6b–h depict the progressive adjustment of neighboring robots as they modify their paths to compensate for the lost coverage. This iterative propagation process redistributes the additional workload among the remaining robots, ultimately achieving a balanced state. The final stage demonstrates the completion of the redistribution process, with all robots evenly sharing the coverage tasks. These results highlight the robustness of the proposed method in managing unexpected robot failures while maintaining efficient and balanced coverage.

**Figure 7:**
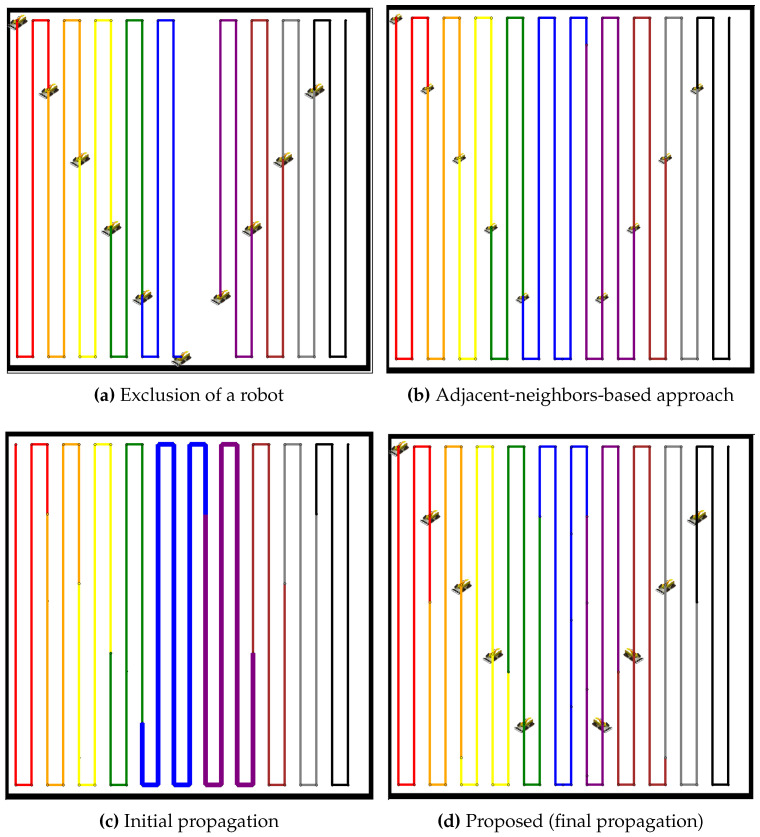
Comparative redistribution results with nine robots after the exclusion of one robot. (**a**) Exclusion of a robot, resulting in a coverage gap. (**b**) Redistribution using the adjacent-neighbors-based approach. (**c**) Initial propagation stage of the proposed method. (**d**) Final propagation stage of the proposed method.

**Figure 8:**
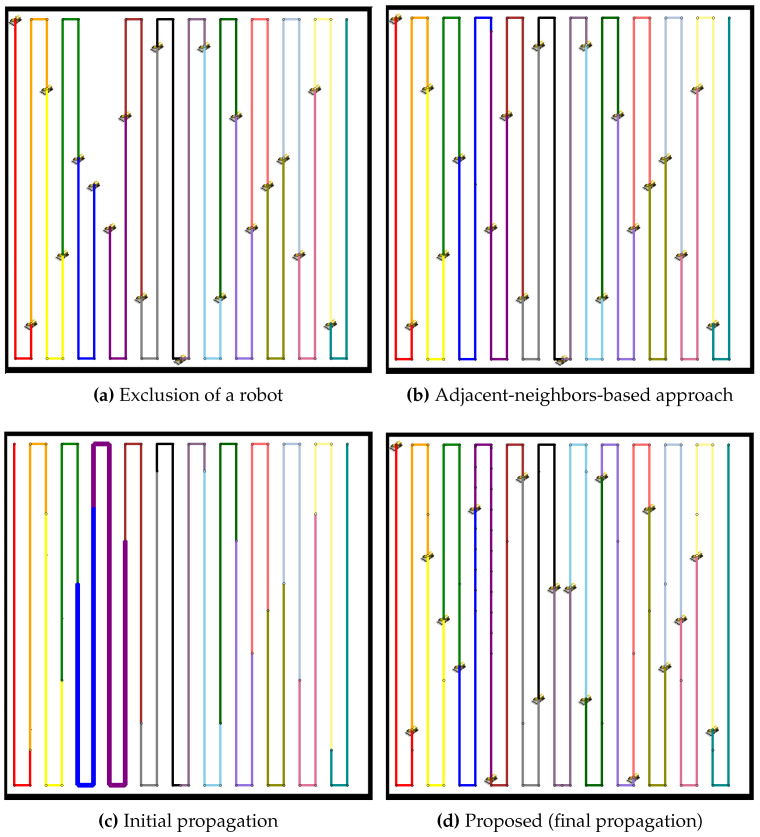
Comparative redistribution results with 19 robots after the exclusion of one robot. (**a**) Exclusion of a robot, leaving a coverage gap. (**b**) Redistribution using the adjacent-neighbors-based approach. (**c**) Initial propagation stage of the proposed method. (**d**) Final propagation stage of the proposed method.

In Figure 7, the proposed method is compared to an adjacent-neighbors-based approach for redistributing the coverage tasks after a robot failure. The exclusion of the robot (see Figure 7a) causes an imbalance in the coverage distribution. The adjacent-neighbors-based approach (see Figure 7b) reallocates the tasks but fails to achieve a balanced workload, as evident from the uneven coverage paths. However, the initial propagation stage (see Figure 7c) of the proposed method shows neighboring robots extending their paths to cover the gap. By the final propagation stage (see Figure 7d), the coverage paths are balanced, ensuring that all robots share the tasks equally, highlighting the efficiency of the proposed method over the adjacent-neighbors-based approach. Similar to the analysis of Figure 7, Figure 8 presents a comparison for a larger team of 19 robots. The exclusion of a robot (see Figure 8a) creates a coverage gap, and the adjacent-neighbors-based approach (see Figure 8b) results in a suboptimal distribution of tasks. The initial propagation stage (see Figure 8c) shows that the proposed method begins to distribute the additional workload more effectively than the adjacent-neighbors-based approach. The final propagation stage (see Figure 8d) demonstrates that the proposed method achieves a balanced distribution, reducing the workload variance and maintaining coverage efficiency across the robot team.

Table 1 summarizes the workload balancing results after applying the proposed propagation-based path redistribution method, along with a comparison to the adjacent-neighbors-based approach. The standard deviation of workload imbalance, $\sigma_{\mathrm{balancing}}$, quantifies the differences in coverage distribution among the robots. After the first propagation, $\sigma_{\mathrm{balancing}}$ remains relatively high (166,113.58 for N=10, 29,490.88 for N=20), reflecting an initial imbalance due to the abrupt reallocation of tasks following a robot failure. However, after the final propagation stage, this imbalance is significantly reduced, dropping to 0 for N=10 robots and 0.97 for N=20, demonstrating the effectiveness of the proposed method. In contrast, the adjacent-neighbors-based approach results in higher imbalance values (169,400.0 for N=10 and 23,072.55 for N=20), highlighting the superior performance of the propagation-based approach in achieving a more balanced coverage distribution.

#### 5.3.2. Environments with Two Obstacles (Map 2)

**Figure 9:**
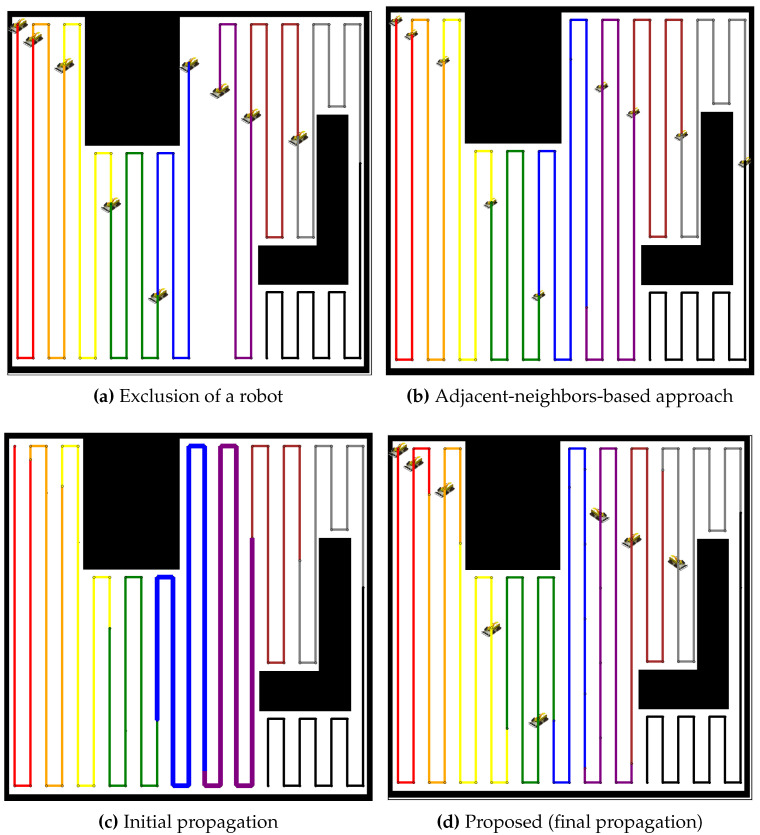
Comparative redistribution results for 9 robots in a map with two obstacles. (**a**) Exclusion of a robot, leading to a coverage gap. (**b**) Redistribution using the adjacent-neighbors-based approach, resulting in an uneven coverage distribution. (**c**) Initial propagation stage of the proposed method, where the workload starts to balance. (**d**) Final propagation stage of the proposed method, achieving balanced coverage across all robots.

In the environment with two obstacles, the proposed method is compared to the adjacent-neighbors-based approach for redistributing the coverage tasks after a robot failure, as shown in Figure 9. The exclusion of the robot causes an imbalance in the coverage distribution, as shown in Figure 9a. The adjacent-neighbors-based approach, as shown in Figure 9b, reallocates the tasks but fails to achieve a balanced workload, as evident from the uneven coverage paths. However, the initial propagation stage, as shown in Figure 9c, of the proposed method shows a significant improvement, with neighboring robots extending their paths to cover the gap. By the final propagation stage, as shown in Figure 9d, the coverage paths are balanced, ensuring that all robots share the tasks equally, highlighting the efficiency of the proposed method over the adjacent-neighbors-based approach.

**Figure 10:**
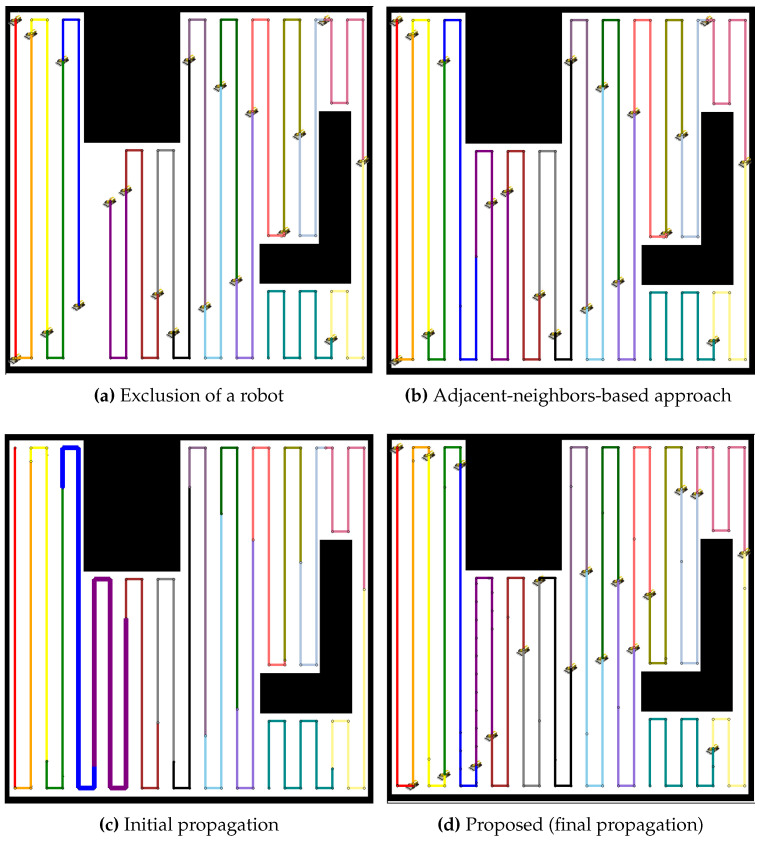
Comparative redistribution results for 19 robots after the exclusion of 1 robot. (**a**) Exclusion of a robot, creating a coverage gap. (**b**) Redistribution using the adjacent-neighbors-based approach, resulting in imbalanced workload distribution. (**c**) Initial propagation stage of the proposed method, where redistribution begins. (**d**) Final propagation stage of the proposed method, achieving balanced workload distribution across all remaining robots.

A similar analysis is applied to a larger team of 19 robots, as presented in Figure 10. The exclusion of a robot, as shown in Figure 10a, creates a coverage gap, and the adjacent-neighbors-based approach, as shown in Figure 10b, results in a suboptimal distribution of tasks. The initial propagation stage, as shown in Figure 10c, demonstrates that the proposed method begins to distribute the additional workload more effectively than the adjacent-neighbors-based approach. The final propagation stage, as shown in Figure 10d, illustrates that the proposed method achieves a balanced distribution, reducing the workload variance and maintaining coverage efficiency across the robot team.

Table 2 compares the balancing results of the proposed propagation-based path redistribution method. The adjacent-neighbors-based approach results in high workload imbalances (128,640.44 for N=10, 17,524.39 for N=20), while the initial propagation still shows significant imbalance. However, by the final propagation stage, the proposed method reduces the imbalance drastically (0.17 for N=10, 0.23 for N=20), demonstrating its effectiveness in achieving equitable workload distribution and maintaining coverage efficiency, even in environments with obstacles and robot failure.

#### 5.3.3. Environments with Multiple Obstacles (Map 3)

**Figure 11:**
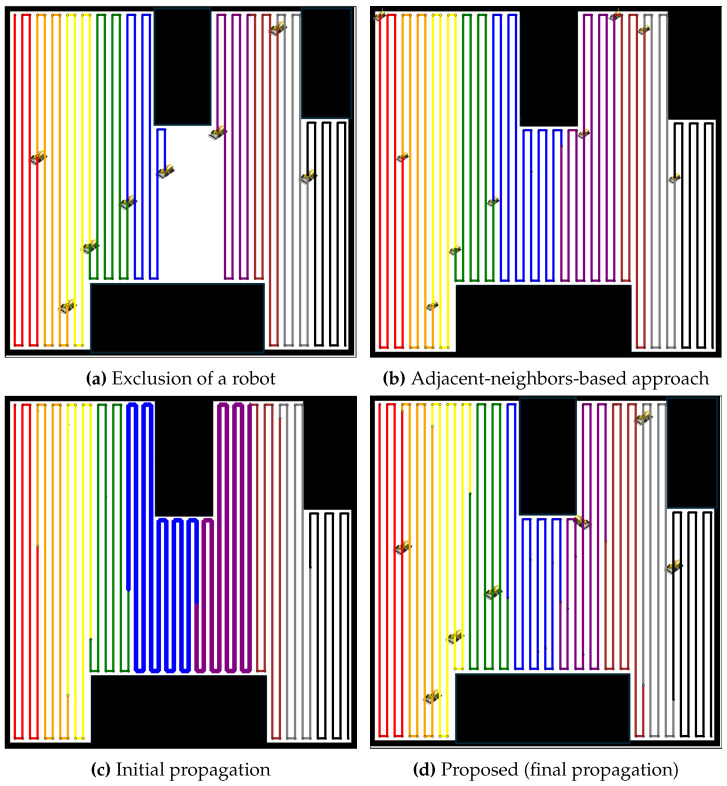
Comparative redistribution results for nine robots after the exclusion of one robot. (**a**) Exclusion of a robot, leading to a coverage gap. (**b**) Redistribution using the adjacent-neighbors-based approach, resulting in uneven workload distribution. (**c**) Initial propagation stage of the proposed method, where redistribution begins. (**d**) Final propagation stage of the proposed method, achieving balanced workload distribution.

The environment with multiple obstacles poses significant challenges for multi-robot coverage. Initially, robots are assigned efficient coverage paths by the BCD algorithm (Figure 4c and Figure 5c). When a robot fails, a coverage gap appears, particularly in constrained areas (Figure 11b). The adjacent-neighbors-based approach attempts redistribution but struggles with uneven task distribution and inefficiencies (Figure 11b). In contrast, the proposed method shows adaptability from the initial propagation stage, with neighboring robots adjusting dynamically to maintain continuous coverage (Figure 11c). By the final propagation stage, the method successfully balances the workload (Figure 11d), handling both open areas and complex environments.

**Figure 12:**
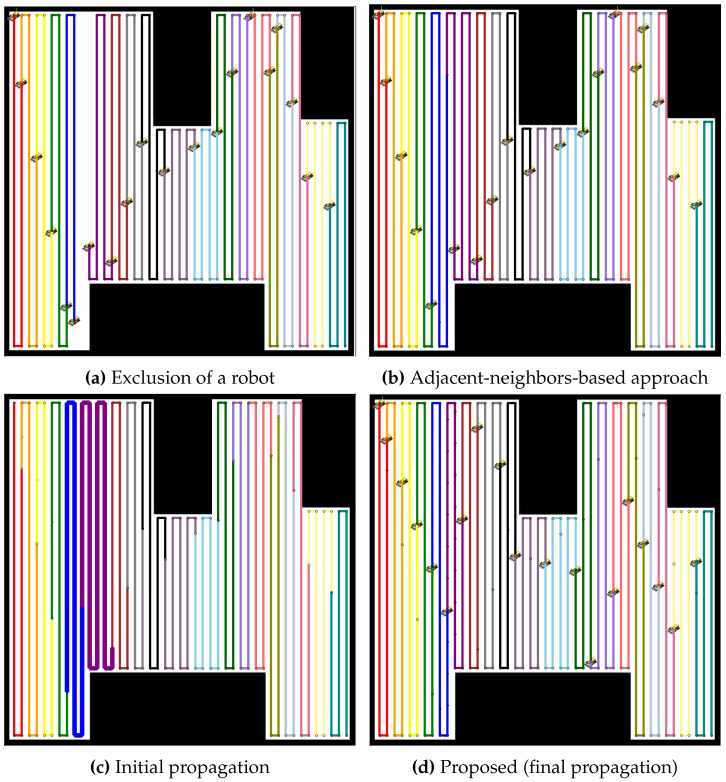
Comparative redistribution results for 19 robots after the exclusion of 1 robot. (**a**) Exclusion of a robot, leading to a coverage gap. (**b**) Redistribution using the adjacent-neighbors-based approach, resulting in uneven workload distribution. (**c**) Initial propagation stage of the proposed method, where imbalances start to be addressed. (**d**) Final propagation stage of the proposed method, achieving an evenly distributed workload.

A similar analysis with 19 robots (Figure 12) shows that after a robot failure, the adjacent-neighbors-based approach results in suboptimal distribution (Figure 12b). The proposed method quickly addresses imbalances during propagation, leading to an evenly distributed workload by the final stage, as shown in Figure 12c,d. This demonstrates the method’s robustness in complex environments with larger teams.

Table 3 summarizes the workload balancing results after applying the proposed propagation-based path redistribution method in environments with multiple obstacles. The standard deviation of workload imbalance, $\sigma_{\mathrm{balancing}}$, highlights the effectiveness of task distribution. In the adjacent-neighbors-based approach, $\sigma_{\mathrm{balancing}}$ remains high (443,814.0 for N=10 and 60,459.49 for N=20), indicating significant imbalance. Although the initial propagation stage of the proposed method still shows considerable imbalance, further adjustments are necessary as $\sigma_{\mathrm{balancing}}$ remains high. However, after the final propagation stage, the imbalance is almost eliminated ($\sigma_{\mathrm{balancing}}$ of 0.69 for N=10 and 0.64 for N=20). These results demonstrate the proposed method’s robustness in balancing tasks efficiently, even in challenging scenarios where conventional methods fail to perform adequately.

#### 5.3.4. Environments with Multiple Lanes (Map 4 and Map 5)

**Figure 13:**
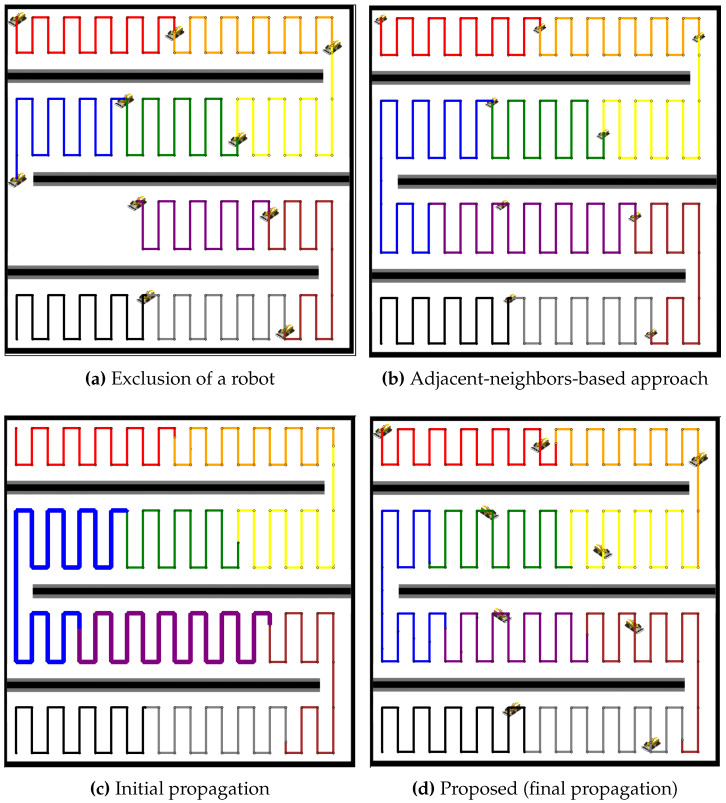
Comparative redistribution results for nine robots after the exclusion of one robot. (**a**) Exclusion of a robot, leading to a coverage gap. (**b**) Redistribution using the adjacent-neighbors-based approach, showing an uneven task distribution. (**c**) Initial propagation of the proposed method, with some improvement but imbalance persisting. (**d**) Final propagation stage of the proposed method, achieving a balanced workload.

The performance of the proposed propagation-based path redistribution method is rigorously evaluated in environments with multiple lanes and compared to the adjacent-neighbors-based approach. Figure 13 illustrates the impact of a robot failure in such a scenario. In Figure 13a, the failure of a robot creates a noticeable gap in the coverage, interrupting the smooth execution of tasks. The adjacent-neighbors-based approach, shown in Figure 13b, attempts to mitigate this issue by redistributing tasks, but fails to effectively balance the workload. This approach overlooks the disparity in path lengths, resulting in uneven coverage path allocation and suboptimal coverage.

Conversely, the initial propagation of the proposed method, as shown in Figure 13c, shows some improvement as neighboring robots begin adjusting to cover the unoccupied coverage area, though imbalance still remains. By the final propagation stage (Figure 13d), the workload becomes well-balanced, with all robots contributing equally. This emphasizes the proposed method’s ability to achieve efficient coverage, even in lane-constrained environments where conventional approaches struggle to perform effectively.

**Figure 14:**
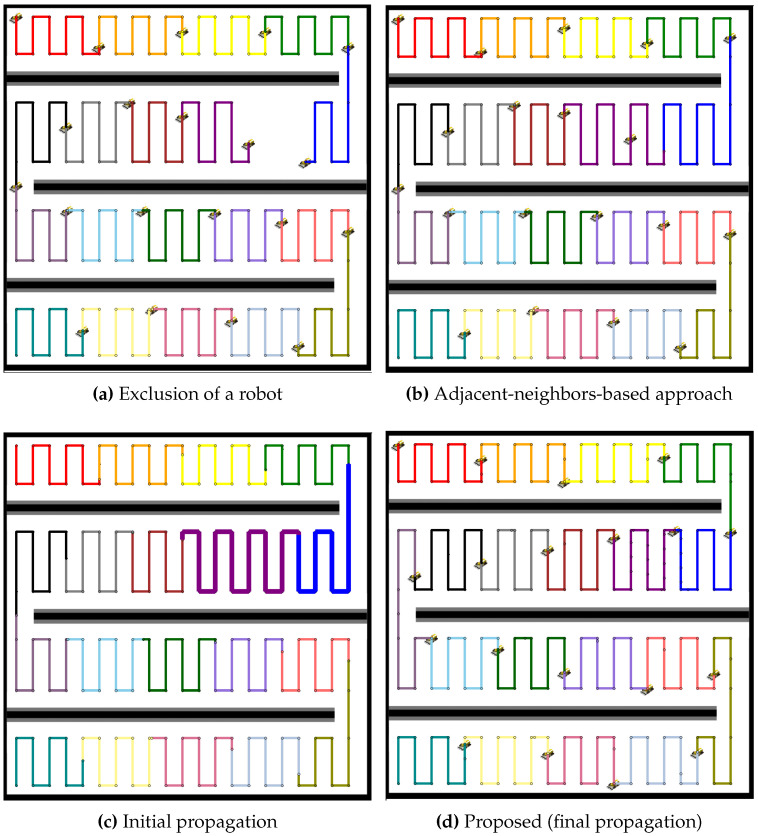
Comparative redistribution results for 19 robots after the exclusion of 1 robot. (**a**) Exclusion of a robot, leading to a coverage gap. (**b**) Redistribution using the adjacent-neighbors-based approach, resulting in uneven task distribution. (**c**) Initial propagation of the proposed method, showing partial improvement but imbalance remaining. (**d**) Final propagation stage of the proposed method, achieving balanced task distribution.

Similarly, in the case of 19 robots (Figure 14), the adjacent-neighbors-based approach fails to balance the tasks effectively, while the proposed method successfully redistributes the paths more efficiently.

**Figure 15:**
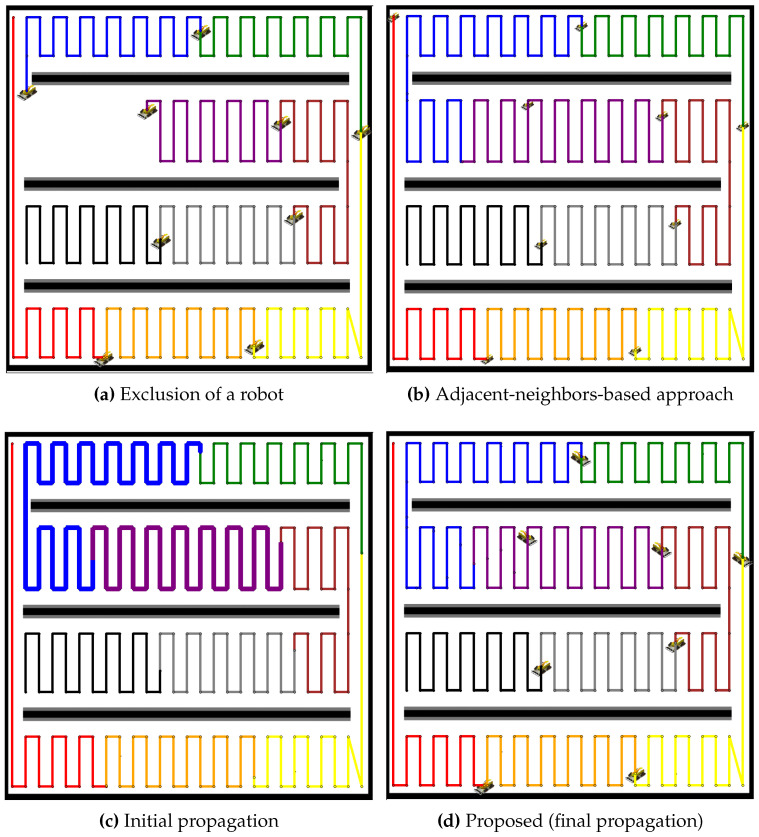
Comparative redistribution results for 9 robots in an environment with multiple segmented areas. (**a**) Exclusion of a robot, resulting in a coverage gap. (**b**) Redistribution using the adjacent-neighbors-based approach, showing uneven task distribution. (**c**) Initial propagation of the proposed method, showing some improvement but still imbalanced. (**d**) Final propagation stage of the proposed method, achieving a well-balanced workload.

In Figure 15, another experiment is conducted in a multiple lane environment. For the case of nine robots, the failure of one robot creates an immediate gap in the coverage, as shown in Figure 15a. The adjacent-neighbors-based approach, illustrated in Figure 15b, attempts to reassign the tasks but fails to balance the workload, as evidenced by the uneven path distribution. The initial propagation phase, depicted in Figure 15c, shows slight improvement, with neighboring robots taking on the coverage of the failed robot. However, it is in the final propagation phase, shown in Figure 15d, where the full effectiveness of the proposed method becomes clear. The coverage paths extend evenly across the environment, ensuring that the remaining robots share the workload equally, maintaining optimal coverage and efficiency.

**Figure 16:**
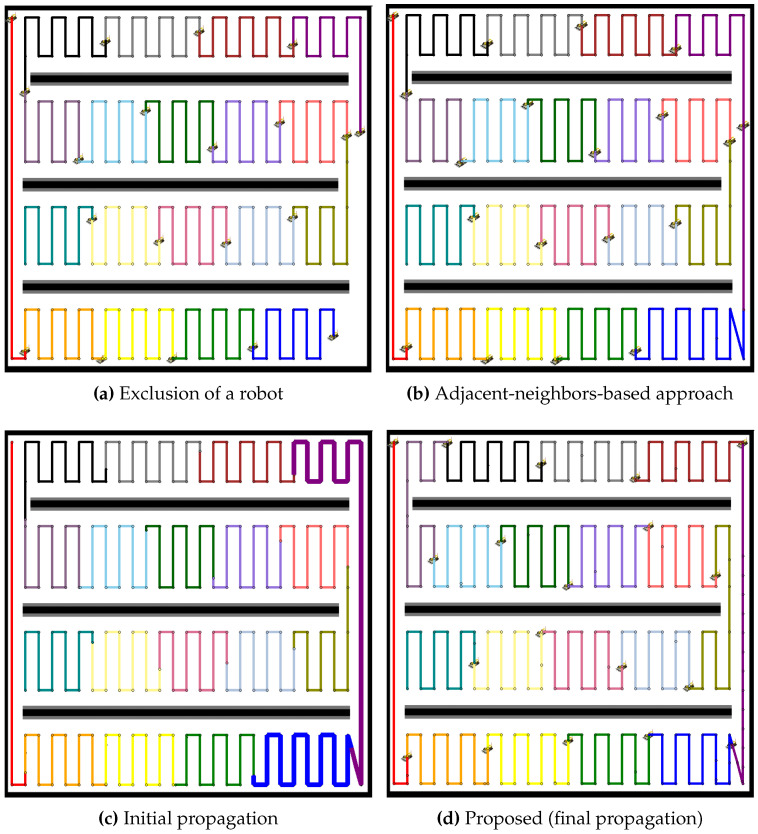
Comparative redistribution results for 19 robots in an environment with multiple segmented areas. (**a**) Exclusion of a robot, creating a coverage gap. (**b**) Redistribution using the adjacent-neighbors-based approach, leaving some robots overburdened. (**c**) Initial propagation of the proposed method, beginning to smooth out the imbalance. (**d**) Final propagation stage of the proposed method, resulting in an evenly distributed workload.

In the case of 19 robots, as shown in Figure 16, the impact of a single robot failure is similarly observed, as depicted in Figure 16a. The adjacent-neighbors-based approach, represented in Figure 16b, again fails to adequately redistribute the tasks, leaving some robots overburdened while others remain underutilized. The initial propagation phase, shown in Figure 16c, begins to smooth out the imbalance, but it is not until the final propagation phase, in Figure 16d, that the proposed method achieves a well-balanced redistribution. The result is a more evenly distributed workload. Both figures highlight the significant advantages of the proposed propagation-based redistribution method over the adjacent-neighbors-based approach. By allowing for dynamic task redistribution, the proposed method not only restores coverage in the affected areas but also ensures that the workload remains balanced across all operational robots, even in the multiply segmented environments.

The balancing analysis in Table 4 demonstrates the superiority of the proposed propagation-based method in reducing workload imbalance compared to the adjacent-neighbors-based approach. For both Map 4 and Map 5, the adjacent-neighbors-based approach results in high imbalances, with $\sigma_{\mathrm{balancing}}$ values of 84,242.89 and 146,288.44 for 10 robots, and 11,479.92 and 19,883.43 for 20 robots, respectively. While the initial propagation phase still shows significant imbalances, the final propagation phase drastically improves balance, achieving near-optimal $\sigma_{\mathrm{balancing}}$ values of 0.44 for Map 4 and 0.57 for Map 5 with 10 robots, and 0.62 for Map 4 and 0.85 for Map 5 with 20 robots. These results clearly highlight the effectiveness of the proposed method in achieving a balanced distribution of tasks across different environments and robot team sizes.

## 6. Discussion

**Figure 17:**
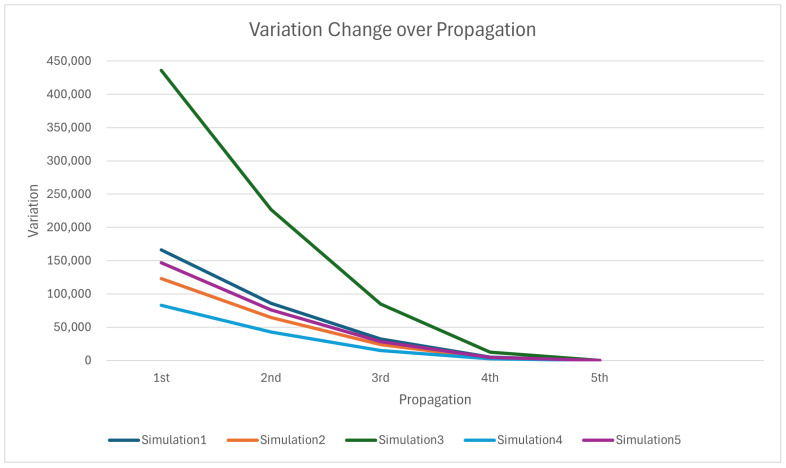
Variance in coverage path lengths according to the number of propagation executions for overall simulations.

The simulation results clearly demonstrate the effectiveness and adaptability of the proposed MCPP and redistribution method, especially in handling robot failures across various environments. As shown in Table 1, Table 2, Table 3 and Table 4, the significant reduction in the value of $\sigma_{\mathrm{balancing}}$, by an average of $1.74\times 10^{-5}$, demonstrates the ability of the proposed method to maintain balanced coverage in the presence of failures. The variance change in coverage path lengths, depicted in Figure 17, further emphasizes this point. Each propagation step results in a steep reduction in variance, indicating that the coverage imbalance is quickly minimized. Initially, the variance is high due to the sudden failure of a robot and the resulting workload imbalance. However, as the proposed propagation-based method progresses, the variance steadily decreases, reaching very low levels by the final propagation stage.

This dynamic redistribution strategy proves robust across diverse environments, ranging from simple open spaces to complex, obstacle-laden scenarios. The proposed method ensures that operational robots adapt their paths to cover the gaps left by failed robots, thus maintaining the mission objectives without significant efficiency loss. Additionally, the propagation-based approach redistributes tasks proportionally among the remaining robots, preventing any single unit from being overburdened. This enhances the system’s scalability and applicability to real-world use cases, such as agricultural fields, warehouse aisles, or long-term surveillance operations.

Moreover, the proposed method demonstrates its ability to execute in a short time, making it feasible for real-time applications. The simulation was performed on a PC with the following specifications: Intel i7 10700K CPU, 16 GB DDR4 RAM (3200 MHz), and Nvidia RTX 3060 GPU. The algorithms were developed using Python 3.8.10, with the GUI implemented through TKinter. Two key metrics were measured during the simulations. $T_{\mathrm{MCPP}}$ is the execution time required for the MCPP, which generates the initial coverage paths for all robots. It represents the time taken to compute optimal paths in a given environment before any robot failures occur. $T_{\mathrm{Propagation}}$ is the execution time for one propagation cycle, which is the core of the proposed method. After a robot failure, the propagation mechanism redistributes the failed robot’s coverage tasks to the neighboring robots. $T_{\mathrm{Propagation}}$ measures how quickly this redistribution occurs. The method’s success depends heavily on minimizing $T_{\mathrm{Propagation}}$ to ensure minimal disruption to the coverage task. As shown in Table 5, the average execution time for MCPP ($T_{\mathrm{MCPP}}$) across various maps was approximately 2.94 s for 10 robots and 2.96 s for 20 robots. The propagation phase, which handles task reallocation after a robot failure, required only 0.0148 to 0.0158 s per iteration ($T_{\mathrm{Propagation}}$). This indicates that the proposed method can quickly redistribute tasks even in complex environments, making it well suited for dynamic, real-world operations.

## 7. Conclusions

This study has demonstrated the robustness and adaptability of the proposed BCD-based Multi-Robot Coverage Path Planning (MCPP) method with dynamic task redistribution, particularly in scenarios where robot failures occur. The proposed propagation-based strategy effectively redistributes the coverage tasks of failed robots to neighboring units, ensuring minimal disruption and maintaining balanced workload distribution across the remaining operational robots. The simulation results across five distinct environments, ranging from simple open areas to complex, obstacle-rich spaces, validate the method’s ability to handle diverse and dynamic environments. The key advantage of this method lies in its dynamic propagation approach, which progressively adjusts the coverage areas of the remaining robots to maintain balance and efficiency. This approach significantly reduces the variance in workload distribution, ensuring that no single robot is overburdened, even as others fail. Our simulations show that the proposed method outperforms conventional methods, such as the nearest neighbors-based approach, in terms of workload variance ($\sigma_{\mathrm{balancing}}$) achieving values below 1, which indicates effective balanced partitioning. In addition, the method’s fast execution time and minimal propagation cycles make it suitable for real-time applications, providing a promising solution for missions that require continuous and reliable monitoring. In our tests, each propagation execution requires less than 0.0158 s per iteration ($T_{\mathrm{Propagation}}$).

Future research should aim to enhance the scalability of the method for larger robot teams, improve energy-efficient path planning, and strengthen inter-robot communication for better coordination in complex missions, while real-world testing in environments with communication delays and energy constraints will provide insights into its practicality and resilience for broader field applications.

## Figures and Tables

**Table 1:** Workload balancing analysis for different approaches in Map 1.

Approach	$\sigma_{\mathrm{balancing}}$
Adjacent-neighbors-based approach (N=10)	169,400.0
Initial propagation (N=10)	166,113.58
Proposed (final propagation) (N=10)	0
Adjacent-neighbors-based approach (N=20)	23,072.55
Initial propagation (N=20)	29,490.88
Proposed (final propagation) (N=20)	0.97

**Table 2:** Workload balancing analysis for different approaches in Map 2.

Approach	$\sigma_{\mathrm{balancing}}$
Adjacent-neighbors-based approach (N=10)	128,640.44
Initial propagation (N=10)	130,683.70
Proposed (final propagation) (N=10)	0.17
Adjacent-neighbors-based approach (N=20)	17,524.39
Initial propagation (N=20)	21,943.51
Proposed (final propagation) (N=20)	0.23

**Table 3:** Workload balancing analysis for different approaches in Map 3.

Approach	$\sigma_{\mathrm{balancing}}$
Adjacent-neighbors-based approach (N=10)	443,814.0
Initial propagation (N=10)	450,863.68
Proposed (final propagation) (N=10)	0.69
Adjacent-neighbors-based approach (N=20)	60,459.49
Initial propagation (N=20)	75,733.03
Proposed (final propagation) (N=20)	0.64

**Table 4:** Workload balancing analysis for different approaches in Map 4 and Map 5.

Approach	$\sigma_{\mathrm{balancing}}$ for Map 4	$\sigma_{\mathrm{balancing}}$ for Map 5
Adjacent-neighbors-based approach (N=10)	84,242.89	146,288.44
Initial propagation (N=10)	85,583.36	148,587.81
Proposed (final propagation) (N=10)	0.44	0.57
Adjacent-neighbors-based approach (N=20)	11,479.92	19,883.43
Initial propagation (N=20)	14,321.88	24,875.30
Proposed (final propagation) (N=20)	0.62	0.85

**Table 5:** MCPP and propagation execution times for different maps (unit: sec).

*N*	Metric	Map 1	Map 2	Map 3	Map 4	Map 5	Avg_Time
10	$T_{\mathrm{MCPP}}$	2.91	3.00	2.95	2.96	2.90	2.94
	$T_{\mathrm{Propagation}}$	0.012	0.014	0.015	0.016	0.017	0.0148
20	$T_{\mathrm{MCPP}}$	2.94	3.01	2.96	2.99	2.93	2.96
	$T_{\mathrm{Propagation}}$	0.015	0.015	0.016	0.016	0.017	0.0158

## Data Availability

The raw data supporting the conclusions of this article will be made available by the authors on request.

## References

[1] Lee S. (2023). An Efficient Coverage Area Re-Assignment Strategy for Multi-Robot Long-Term Surveillance. IEEE Access.

[2] Choton J.C., Prabhakar P. Optimal Multi-Robot Coverage Path Planning for Agricultural Fields using Motion Dynamics. Proceedings of the 2023 IEEE International Conference on Robotics and Automation (ICRA).

[3] Lee S., Kim H.J., Lee B.H. (2020). An Efficient Rescue System with Online Multi-Agent SLAM Framework. Sensors.

[4] Galceran E., Carreras M. (2013). A survey on coverage path planning for robotics. Robot. Auton. Syst..

[5] Foster A.J.I., Gianni M., Aly A., Samani H., Sharma S. (2024). Multi-Robot Coverage Path Planning for the Inspection of Offshore Wind Farms: A Review. Drones.

[6] Shah K., Schmidt A., Ballard G., Schwager M. (2022). Large Scale Aerial Multi-Robot Coverage Path Planning. Field Robot..

[7] Almadhoun R., Taha T., Seneviratne L., Zweiri Y. (2019). A survey on multi-robot coverage path planning for model reconstruction and mapping. SN Appl. Sci..

[8] Cai C., Chen J., Yan Q., Liu F. (2023). A Multi-Robot Coverage Path Planning Method for Maritime Search and Rescue Using Multiple AUVs. Remote Sens..

[9] Song J., Gupta S. (2019). CARE: Cooperative Autonomy for Resilience and Efficiency of Robot Teams for Complete Coverage of Unknown Environments under Robot Failures. Auton. Robot..

[10] Choset H., Pignon P. (1998). Coverage path planning: The boustrophedon cellular decomposition. Field and Service Robotics.

[11] Sun R., Tang C., Zheng J., Zhou Y., Yu S. (2019). Multi-robot Path Planning for Complete Coverage with Genetic Algorithms. Proceedings of the Intelligent Robotics and Applications Lecture Notes in Computer Science.

[12] Noh D.K., Choi J.H., Choi J.S., Byun D.S., Kim Y., Kim H.R., Baek S.M., Lee S.H., Myung H. MASS: Multi-Agent Scheduling System for Intelligent Surveillance. Proceedings of the 2022 19th International Conference on Ubiquitous Robots (UR).

[13] Madridano Á., Al-Kaff A., Martín D., de la Escalera A. (2021). Trajectory Planning for Multi-Robot Systems: Methods and Applications. Expert Syst. Appl..

[14] Rekleitis I., New A.P., Rankin E.S., Choset H. (2008). Efficient boustrophedon multi-robot coverage: An algorithmic approach. Ann. Math. Artif. Intell..

[15] Gabriely Y., Rimon E. (2001). Spanning-tree based coverage of continuous areas by a mobile robot. Ann. Math. Artif. Intell..

[16] Tang J., Sun C., Zhang X. MSTC*: Multi-robot coverage path planning under physical constraints. Proceedings of the IEEE International Conference on Robotics and Automation.

[17] Tang J., Ma H. Large-Scale Multi-Robot Coverage Path Planning via Local Search. Proceedings of the AAAI Conference on Artificial Intelligence.

[18] Lu J., Zeng B., Tang J., Lam T.L., Wen J. (2023). TMSTC*: A Path Planning Algorithm for Minimizing Turns in Multi-Robot Coverage. IEEE Robot. Autom. Lett..

[19] Kapoutsis A.C., Chatzichristofis S.A., Kosmatopoulos E.B. (2017). DARP: Divide Areas Algorithm for Optimal Multi-Robot Coverage Path Planning. J. Intell. Robot. Syst..

[20] Karapetyan N., Benson K., McKinney C., Taslakian P., Rekleitis I. Efficient Multi-Robot Coverage of a Known Environment. Proceedings of the IEEE/RSJ International Conference on Intelligent Robots and Systems (IROS).

[21] Azpúrua H., Freitas G.M., Macharet D.G., Campos M.F.M. (2018). Multi-Robot Coverage Path Planning Using Hexagonal Segmentation for Geophysical Surveys. Robotica.

[22] Huang X., Sun M., Zhou H., Zhang J. (2020). A Multi-Robot Coverage Path Planning Algorithm for the Environment With Multiple Land Cover Types. IEEE Access.

[23] Gong J., Lee S. (2023). Hierarchical Area-Based and Path-Based Heuristic Approaches for Multirobot Coverage Path Planning with Performance Analysis in Surveillance Systems. Sensors.

[24] Zhou Z., Lee D.J., Yoshinaga Y., Balakirsky S., Guo D., Zhao Y. Reactive task allocation and planning for quadrupedal and wheeled robot teaming. Proceedings of the 2022 IEEE 18th International Conference on Automation Science and Engineering (CASE).

[25] Fazli P., Davoodi A., Mackworth A.K. (2013). Multi-robot repeated area coverage. Auton. Robot..

[26] Collins L., Ghassemi P., Esfahani E.T., Doermann D., Dantu K., Chowdhury S. Scalable coverage path planning of multi-robot teams for monitoring non-convex areas. Proceedings of the IEEE International Conference on Robotics and Automation.

[27] Almadhoun R., Taha T., Seneviratne L., Zweiri Y. (2022). Multi-Robot Hybrid Coverage Path Planning for 3D Reconstruction of Large Structures. IEEE Access.

[28] Bähnemann R., Lawrance N., Chung J.J., Pantic M., Siegwart R., Nieto J., Ishigami G., Yoshida K. (2021). Revisiting Boustrophedon Coverage Path Planning as a Generalized Traveling Salesman Problem. Field and Service Robotics.

